# Dermoscopy and skin imaging light sources: a comparison and review of spectral power distribution and color consistency

**DOI:** 10.1117/1.JBO.27.8.080902

**Published:** 2022-08-08

**Authors:** Katharine L. Hanlon, Grace Wei, Lilia Correa-Selm, James M. Grichnik

**Affiliations:** a University of South Florida, Department of Dermatology and Cutaneous Surgery, Tampa, Florida, United States; b Moffitt Cancer Center, Cutaneous Oncology, Tampa, Florida, United States; c University of South Florida, Morsani College of Medicine, Tampa, Florida, United States

**Keywords:** dermoscopy, spectral power distribution, skin imaging, light-emitting diodes, color science, color rendering

## Abstract

**Significance:**

Dermoscopes incorporate light, polarizers, and optical magnification into a handheld tool that is commonly used by dermatologists to evaluate skin findings. Diagnostic accuracy is improved when dermoscopes are used, and some major artificial intelligence (AI) projects have been accomplished using dermocopic images. Color rendering consistency and fidelity are crucial for clinical diagnostics, AI, and image processing applications.

**Aim:**

With many devices available on the market, our objective was to measure the emission spectra of various dermoscopes, compare them with other light sources, and illustrate variations in reflected colors from images of a reference sample.

**Approach:**

A spectrometer measured the spectral power distribution (SPD) produced by four dermoscope models and three alternate light sources, illustrating differences in the emission spectra. Most dermoscopes use light-emitting diodes (LEDs), which are inconsistent when compared with one another. An LED was compared with halogen, xenon-arc, and daylight sources. Images of a micro ColorChecker were acquired from several sources, and three specific colors were selected to compare in CIELAB color space. Color consistency and color fidelity measured by color rendering index (CRI) and TM-30-18 graphical vectors show variation in saturation and chroma fidelity.

**Results:**

A marked degree of variation was observed in both the emission and reflected light coming from different dermoscopes and compared with other sources. The same chromophores appeared differently depending on the light source used.

**Conclusions:**

A lack of uniform illumination resulted in inconsistent image color and likely impacted metamerism and visibility of skin chromophores in real-world settings. Artificial light in skin examinations, especially LEDs, may present challenges for the visual separation of specific colors. Attention to LEDs SPD may be important, especially as the field increases dependency on machine/computer vision.

## 1 Background

### 1.1 Dermoscopy

The practice of dermoscopy has evolved significantly over the past century.^1^ Dermatologists use dermoscopes to visualize skin findings and improve diagnostic accuracy; this practice has steadily grown to be widely accepted. Dermoscopy courses, residency training, algorithms, atlases, working groups, and other academic resources are readily available. There is a broad selection of dermoscopes on the market, each with distinct technical specifications. However, it is uncommon to find the technical specifications, such as spectral power distribution (SPD), color rendering index (CRI), or even Kelvin temperature, made available to users of dermoscopic devices, although this information is accessible for lighting in other sectors.

The clinical utility of dermoscopy has been demonstrated in terms of improved diagnostic accuracy.^2^^,^^3^ Dermoscopy has also been the subject of several high-profile studies on artificial intelligence (AI) and machine learning (ML) approaches to diagnostics.^4^^,^^5^ An important diagnostic criterion is color, both for pigmented lesions and other skin findings.^1^^,^^6^ In fact, color itself can be used as a feature for deep learning algorithms used for AI in dermatology.^7^^–^^10^ Polarized light dermoscopy (technically cross-polarized) has been shown to improve the visibility of colors,^11^^,^^12^ amongst other features. Color consistency, fidelity, reproducibility, and standardization are needed for both clinical diagnostics as well as computer vision and image processing applications.^13^^,^^14^

In the past, halogen bulbs were common,^15^ whereas today most devices make use of light-emitting diodes (LED). The SPD of halogen (tungsten) generates a continuous distribution of light across the visible spectrum, with most of the energy concentrated in the red and infrared portion of the spectrum, giving older dermoscopes their characteristic “warm” light. The Kelvin temperature of dermoscopes may eventually be incorporated into metadata fields available for DICOM attribute descriptors.^16^ Some dermoscopes now integrate multispectral and colored LED, xenon, and UV sources into devices;^17^^–^^20^ however, most dermoscopes use white LEDs. A lack of uniformity in illuminating chromophores contained in skin could potentially reduce diagnostic accuracy.^21^

### 1.2 LED Advances

LED technology itself has grown tremendously in the past two decades, both in terms of science and applications related to medicine^22^ and imaging. In 2014, the invention of the blue LED won a physics Nobel prize. There is considerable and ongoing effort to improve LEDs efficiency, color rendering, and fidelity.^23^ The Nobel committee recognized the challenge of producing blue LEDs, noting its importance to producing white LEDs. Phosphor doping of the blue LED is a common method for producing white light; however, this approach has been shown to produce light that is insufficient for medical lighting in a number of scenarios. In 2000, the surgeon performing the first surgery using LED loupes noted that it was difficult to distinguish arteries from veins.^24^ The two main issues with phosphor-conversion of LEDs are that energy is lost when photons are converted from shorter to longer (i.e., blue to red), and some of the energy is wasted outside the visible spectrum.^25^ To make “good” white light, it should match (or generate metamers of) daylight. The chromaticity of the light source is best judged side-by-side with other light sources, and depending on SPD, it may appear as “white” or having a “tint.” Practically, the chromaticity of an LED may not be “white,” although this was the manufacturer’s intention. There is an ongoing effort to improve spectral matching for LEDs;^26^ however, the issue of colors rendering inconsistently was evident in our experiment.

### 1.3 Color Science

Great advances have been achieved since early color theories such as those described by Newton as far back as the 1700s. Both SPD and color reflection can be measured quite precisely; however, despite these ever-advancing technical solutions for color vision, it is not possible to ensure that two individuals perceive the same colors identically, regardless of the accuracy of measurement devices.^27^

The International Commission on Illumination (CIE) defines color spaces and formulas for measurement and develops reference standards and indexes, among many other functions.^28^^,^^29^ The CIE defines color rendering as “the effect of an illuminant on the color appearance of objects by conscious or subconscious comparison with the color appearance under a reference illuminant.” The CIE categorizes illuminants into series, having designations for halogen (A), daylight (D) series, fluorescent series (F), and a newer addition of LED series. There have been some efforts to match the SPD of LED more precisely to other color spaces and standard illuminants;^26^^,^^30^ this research reinforces that it may not be possible to manufacture a LED that perfectly matches a desired SPD, although much progress has been made to reduce spectral mismatching of LED. The goal of reviewing LEDs in dermoscopes is not to determine a perfect white LED for skin illumination, but rather to highlight differences in rendering ability compared with one another and when compared with standard illuminants.

The common CRI is a helpful, but somewhat limited, system for the measurement of color. This is because CRI is known to be prone to manipulation or “gaming” in which metamerism is leveraged to improve rendering values. Further, the values do not always correlate well to visual evaluation, prompting the development of additional fidelity indexes aimed at improving the range in scientific use.^31^

Developed by the Illuminating Engineering Society, TM-30-18 measures fidelity and chroma of light; it was developed as an alternative reference to CRI for increased accuracy.^32^ This index includes an expanded reference that recently was shown to outperform other evaluation criteria when “naturalness” is the most important aspect.^33^

Some research has suggested that color accuracy or the perceptual rendering of colors as they appear to the human eye is not necessarily the crucial aspect for visualization of specific features under dermoscopy;^34^ however, this research did not compare the degree of differences, nor did it address how two different colors might appear identical when color fidelity is suboptimal. It is feasible that the observation by Avanaki et al. of “less colorful images enhancing visualization of certain details” actually describes ancillary (but important) aspects of feature perception as a function of simultaneous contrast and conspicuity. Simultaneous contrast in both color and greyscale images may reduce feature conspicuity, a well-described phenomenon in the field of radiology.^35^^,^^36^ A number of publications appear to dispute the claim that perceptual rendering of color is inconsequential in dermoscopy images,^13^^,^^37^^,^^38^ with one notable publication^39^ actually comparing the features that are perceptible from different dermoscopes and illustrating how some light can completely obfuscate specific findings. Additionally, the ability of ML algorithms to leverage color data^9^^,^^10^ may rely upon consistency and rendering capability.

Measuring differences in color and limiting the tolerance for these differences can be described with the use of DeltaE2000 (ΔE): the Euclidean distance between two colors in a given color space. The most recent color difference formula is the CIEDE2000^40^ (Fig. S2 in Supplemental Material). Generally, the perceptible ΔE difference between two colors that is noticeable is 1, with opposite colors at 100 (e.g., green and red). An acceptable difference in color, or tolerance, for industrial, commercial, or entertainment industries applications may indeed be wider or more permissive than those in science and medicine. The tolerance of a given application may be in the range of one to seven, with greater perceptible differences having a higher number. No standard tolerance for acceptable color difference has yet been defined for skin imaging; however, it is thought to lie between two and four in the field of dentistry^41^^,^^42^ as color pertains to matching dental ceramics. The difference in colors perceived in skin is arguably more critical than the color difference in teeth, considering the purpose of matching colors in dentistry is primarily cosmetic in nature, whereas the colors in skin imaging may have diagnostic significance. In the field of skin imaging and skin color matching, various approaches to quantify color have been used,^43^ with some use of novel facial color contrast measurements.^44^ Spectrophotometers are used for some skin color variation research, which employs pulsed xenon-arc light. In manufacturing and printing, greater degrees of LED differences can reduce the aesthetic appeal of an image or product; in science and medicine, a greater difference may indicate device calibration errors, defective equipment, or in our case, a lack of standards.

**Fig. 1:**
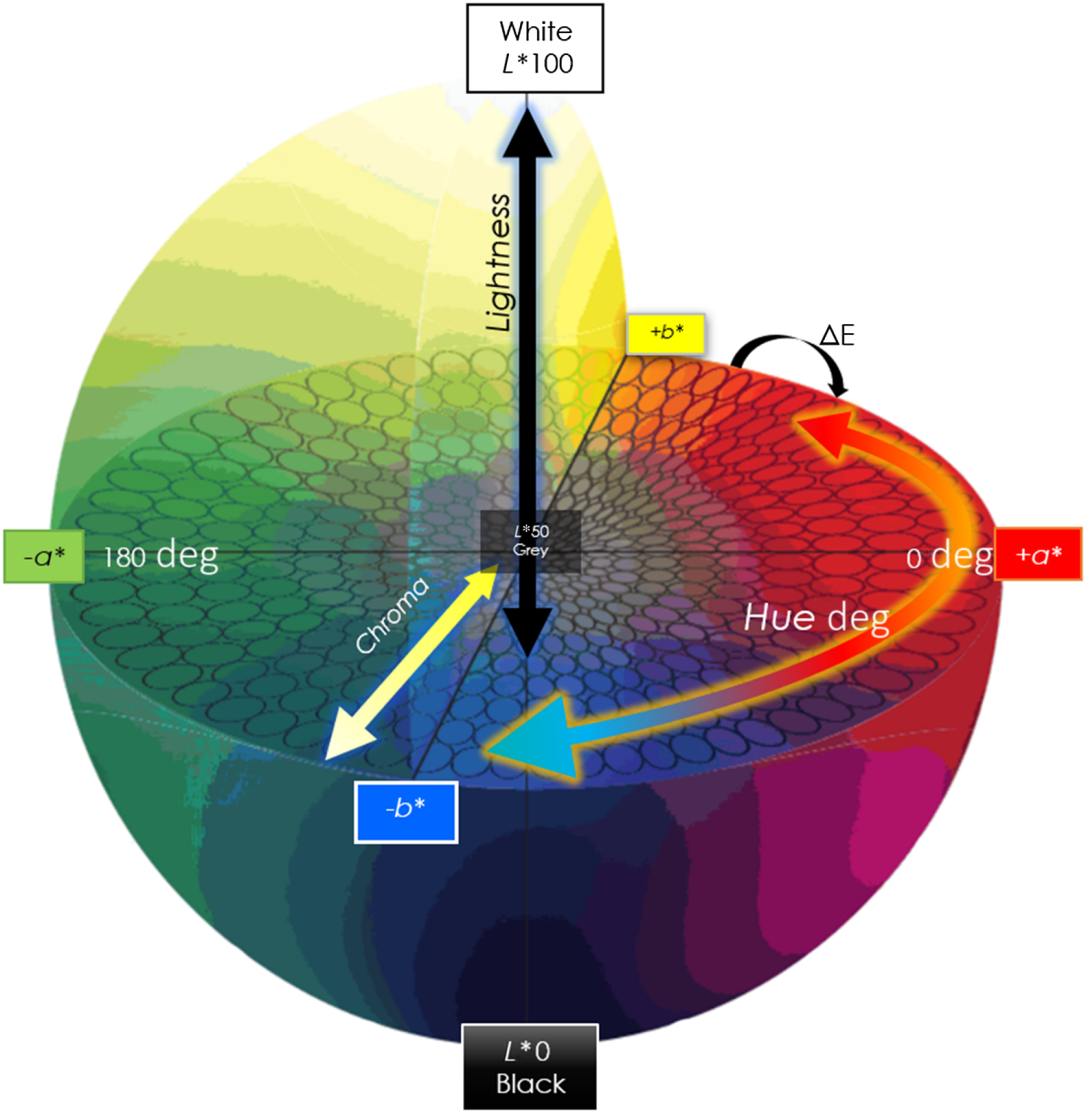
CIELAB or L*a*b* color space with a graphical representation of ΔE. A device-independent, three-dimensional color space that is intended to be perceptually uniform relative to human color vision, such that a given numerical change is correlated to a perception of change in color. L*a*b* values are correlated to lightness, chroma, and hue, where L* ranges between 0 (black) and 100 (white), a* describes the red–green axis, and b* describes the blue–yellow axis, akin to the opponent pair of the human visual system. The distance between a pair of colors is reported at the color difference ΔE. CIELAB and ΔE were developed by the Commission Internationale de l’Eclairage (CIE) with the intent of measuring the Euclidean distance within a given color space. Intended to be a difference calculator, ΔE of 1 represents the smallest noticeable change in color. ΔE is illuminant dependent; a change under differing illuminants illustrates a metamerism index, and two colors that appear the same under differing illuminant sources are considered metamers.

The color space chosen to perform the measurements can improve the ability to quantify perceptual differences, reducing the effects of metamerism. The CIELAB color space (or LAB) is a three-dimensional, device-independent model that is intended to be perceptually uniform and describes relative luminance independently from chroma (Fig. 1).

CIELAB measurements can be correlated to skin color and cutaneous findings, decreasing subjectivity in tasks such as classification of Fitzpatrick skin type.^45^ A reference light source of D50 (also developed by the CIE) is commonly used for color matching applications. D50 is a theoretical light source that closely approximates white light and is considered to be an acceptable industry reference standard.

## 2 Methods

A spectrometer (Sekonic Spectromaster C-800, Tokyo, Japan) was used to measure the SPD of light emitted by four different dermoscopes produced by two manufactures, one with an additional color setting, generating a total of five dermoscope’s emission data. For a frame of reference to compare with other real-world light sources that may be used in dermatology, three additional lights were measured: LED from an iPhone flash, xenon-arc from a Nikon flash, and daylight (at noon). Reflection measurements were made using uncorrected images of a micro ColorChecker (X-rite Inc. Michigan, United States) acquired from four of the devices and two additional light sources (for reference); three colors were selected to be arranged side-by-side for qualitative analysis, and ΔE was noted for two LED devices. Reflection images were acquired with each dermoscope through the lens of a mobile camera, with the dermoscope lens placed directly on the micro ColorChecker using a small piece of foam in a darkened room. For a real-world comparison of how reflected light from different dermoscopes may impact color and ultimately feature perception, two images of a dermal nevus were included for visual comparison side-by-side with their histograms.

### 2.1 Spectral Power Distribution

Spectrometer measurements were taken from (1) Heine (Germany) DELTA10 (halogen), (2) DELTA20T, (3) DELTA30 as well as 3Gen DermLite (California, United States), (4) DL4, and (5) DL4 with Orange Boost. 3Gen’s DL3 and Foto II were also measured (not shown), and the LED SPD matched the other DermLite products.

We measured SPD of three additional sources that may represent cutaneous imaging in real-world settings: Apple iPhone 11 LED flash, xenon-arc (emitted from a Nikon flash), and daylight (southern hemisphere at noon).

Kelvin temperature and CRI were measured for the four dermoscopes and the additional sources. Reference illumination was set to D50 (or CCT 5000 K) for the closest standard approximation to white light.

TM-30 graphical vectors were generated and compared to demonstrate color saturation and chroma fidelity.

### 2.2 Reflection Measurements

ColorChecker images were acquired in DNG format, uncorrected (no white balance settings were applied) using an Apple iPhone 11 set to manual with the same exposure settings for four dermoscopes and two references; see Fig. S1 in Supplemental Material. The ambient light was dimmed prior to the serial acquisition of a micro ColorChecker with each dermoscope, at an exposure setting that was sufficient for the varying intensity of the different dermoscopes. The iPhone camera lens was placed directly against the dermoscopes, or a magnetic adapter was used to secure the device to the phone (when available), and the ColorChecker was placed upon a piece of foam to flatten the image field.

ColorChecker images were set to 5000 K, and L*a*b* values were recorded for specific colors. ColorChecker images of three colors (orange–yellow, blue sky, and light skin) were cropped and placed next to each other for qualitative visual analysis.

ΔE was measured using Bruce Lindblooom’s calculator^46^ for dermoscopes 3 and 4, representing two of the more commonly used current devices. The formula for DeltaE2000 is provided in Fig. S2 in Supplemental Material.

Dermoscopes 3 and 4 were used to acquire two images of a dermal nevus. The exposure settings were matched, and the images were placed side-by-side for qualitative analysis. The images’ histograms, as well as a single L*a*b* measurement value from the same area, are visible in both images (Fig. 7).

## 3 Results

**Fig. 2:**
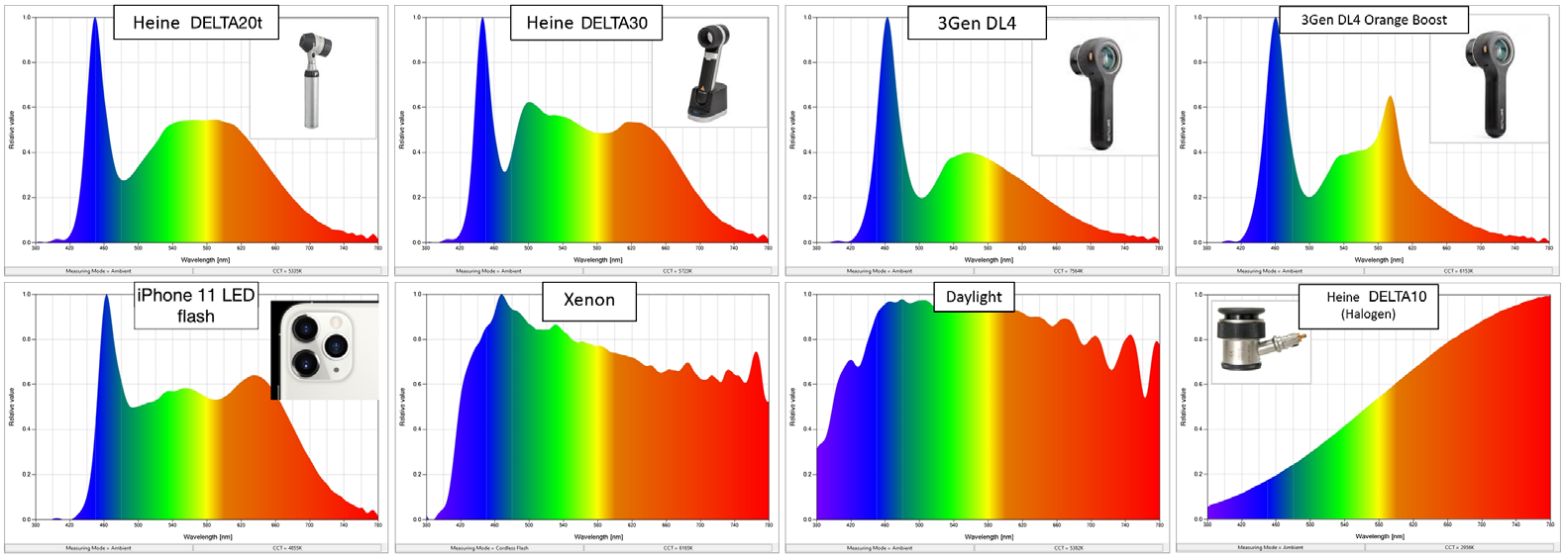
SPD comparison of different light sources. Four^§^ dermoscopes, an Apple iPhone 11, and two alternate reference sources (xenon-arc and daylight) showing marked variations between different types of light and variations of SPD of “white” LEDs used in different dermoscopes. Halogen emits more energy in the red and near-infrared portions of the spectrum, xenon and daylight have broad SPDs, and all LEDs show the characteristic narrow spike in the blue region, a dip in energy produced in the 400- to 500-nm range, and a gradient of lowered energy emitted in the red and near-infrared regions. ^§^Plus one additional setting of “orange boost.”

**Fig. 3:**
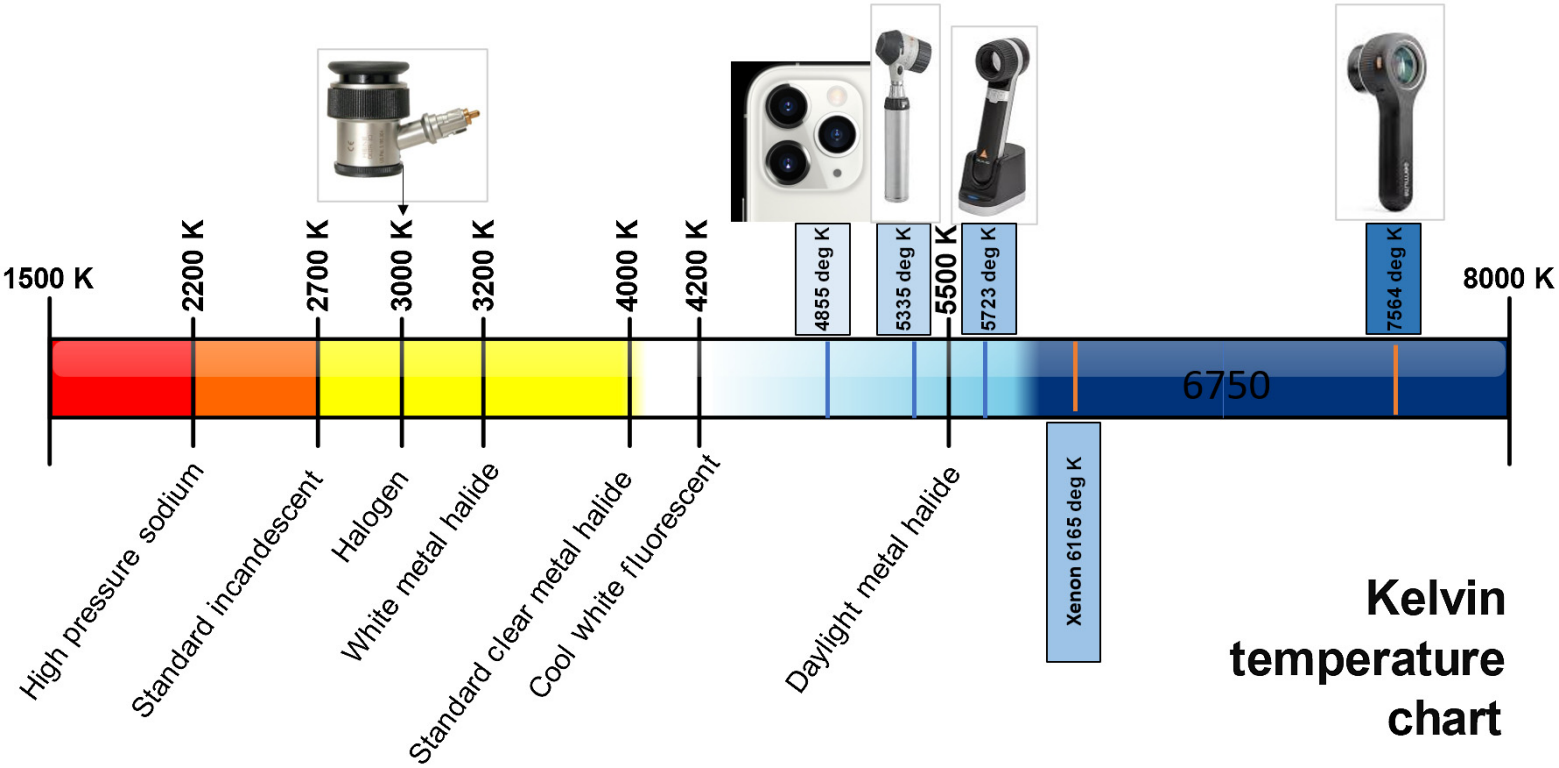
Kelvin temperatures of different LED devices. Correlated color temperature measures degrees Kelvin indicating the different hues of light sources emitted by an idealized, opaque, non-reflective body. Color temperature ranges from warm to cool, along a locus correlating infrared light (at ∼100  K) to ultraviolet light (at ∼50,000  K). Blue occurs at higher Kelvin temperatures, and red occurs at lower Kelvin temperatures, which is the inverse of what is visually expected from “warm/cool” colors. In between the two extremes lies halogen (≈3000  K), daylight (≈5500  K), xenon (≈6200  K), and LEDs (variable K). Shown here are three LED dermoscopes and their relative position on the Kelvin Chart, as well as a halogen dermoscope and the LED from an iPhone for comparison.

Spectrometer measurements showed a high degree of variation in SPD between light sources. In Fig. 2, the relative Kelvin temperatures or correlated color temperatures (CCTs) of the different devices are illustrated on the Kelvin Chart, which indicates the hue of each light source (Fig. 3).

**Fig. 4:**
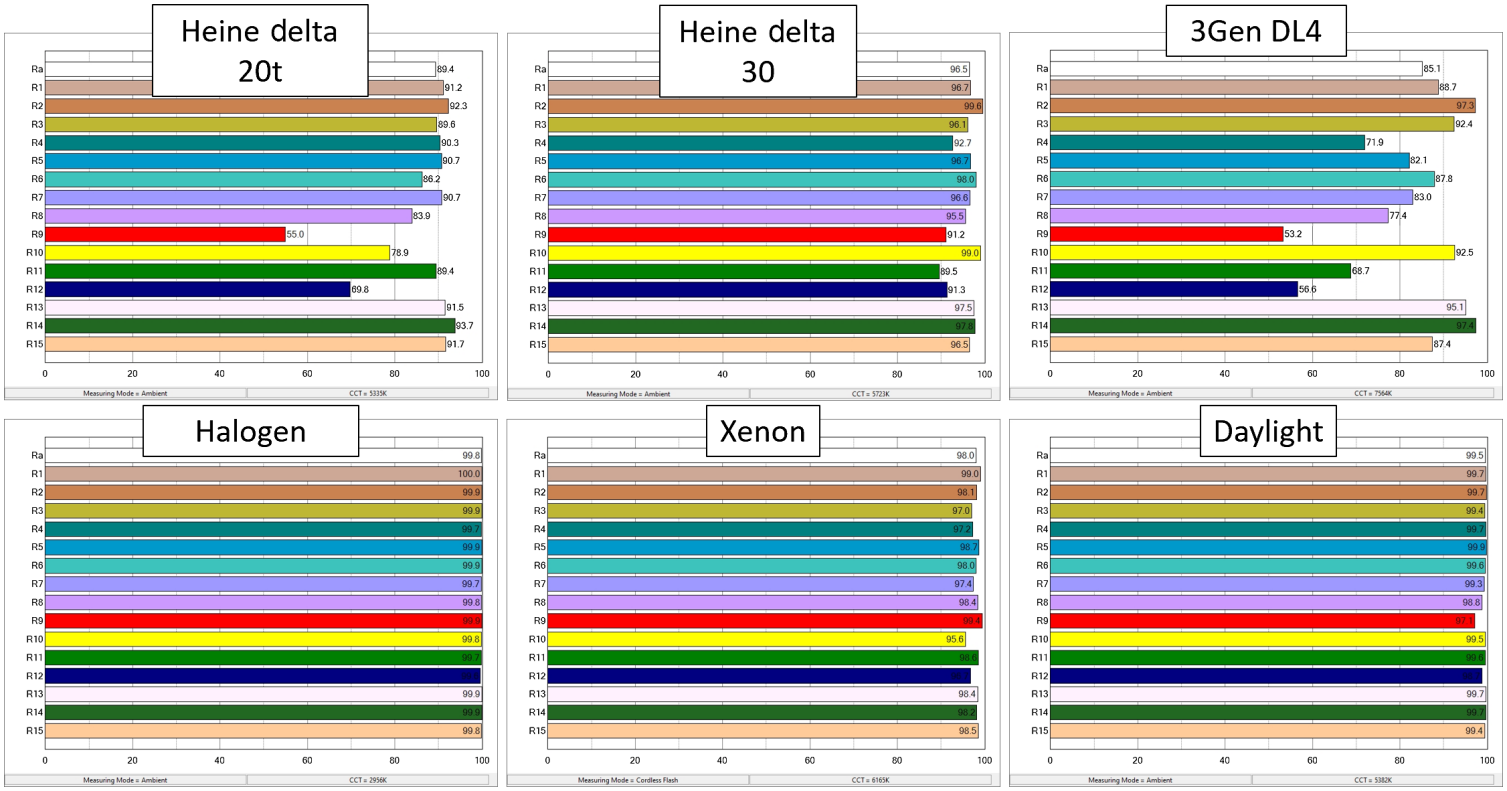
CRI comparison. A quantitative scale of how well a light source is able to reveal the true color of a material, CRI was developed by the CIE. CRI has positive and negative values, but a value of 100 indicates a perfect match to a D65 color point. D65 is meant to simulate the SPD of daylight at ∼6500  K. CRI has fallen out of favor due to it not being a good indicator of visual assessment. Shown here is the CRI of three dermoscopes (white LED) (top row) to three other light sources including halogen, xenon, and daylight (bottom row). Note that the measurement of actual daylight produced lower CRI values than the halogen source, a known limitation of CRI.

**Fig. 5:**
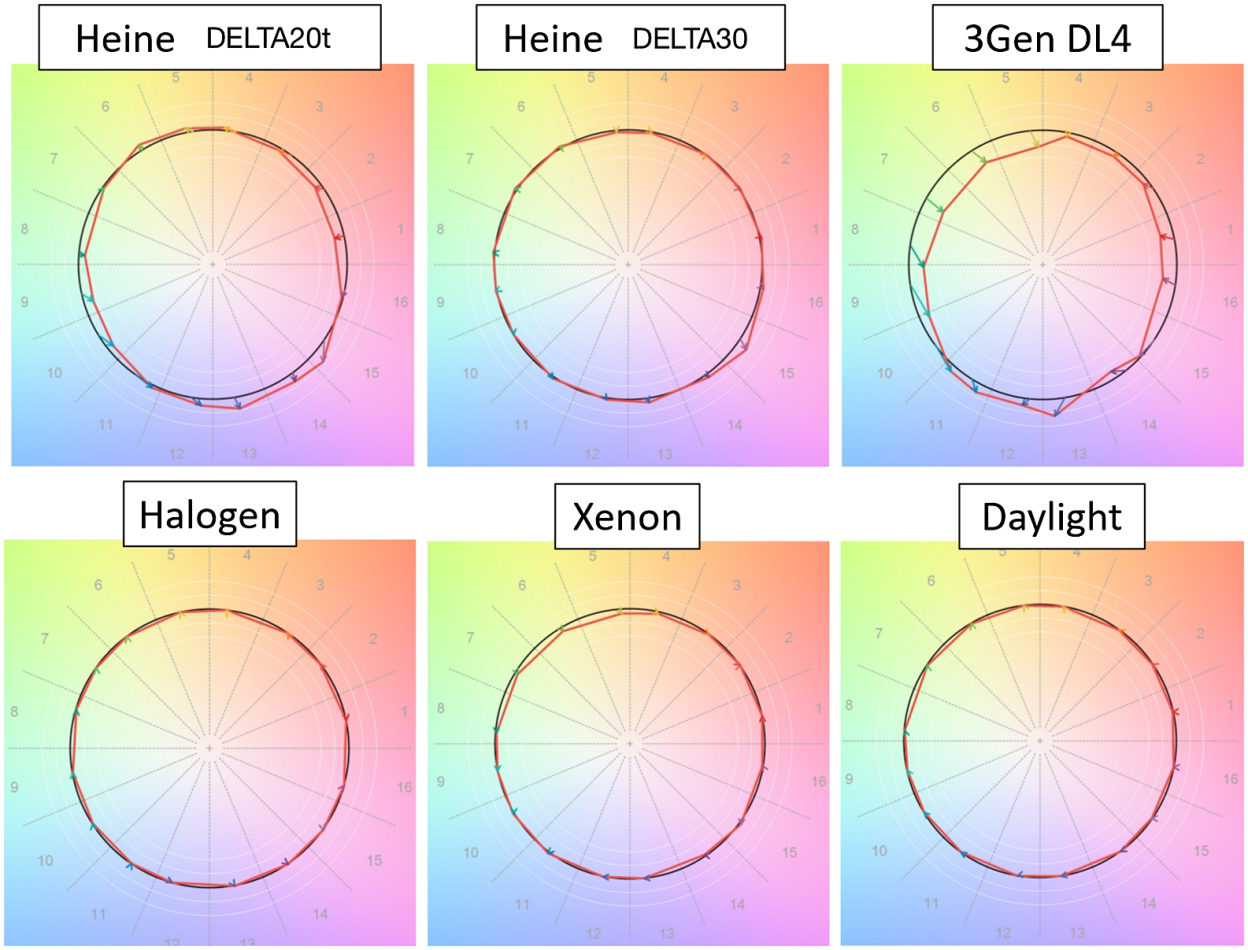
The Technical Memorandum TM-30-18 chroma shift color vectors. Developed by the Illuminating Engineering Society. TM-30 evaluates color rendition, chroma, and gamut of the light sources using a set of 99 color evaluation samples. TM-30 is a reference-based system; here the reference color is set to D65. The chroma shift vectors illustrate both direction and magnitude of change from the reference. On the top row is a comparison of three white LED dermoscopes, and on the bottom row, three other light sources including halogen, xenon, and daylight are shown. Of note, the DL4 illustrates how some colors are rendered undersaturated, such as green, orange, and red, while blue appears oversaturated. TM-30 is analogous to CRI, but it uses more updated color science and represents human perception better. Note that halogen renders colors most similarly to D65.

CRI and TM-30 quantified color consistency and saturation levels for the dermoscopes tested (Figs. 4 and 5). Of the LED dermoscopes, #3 produced results closest to reference standard D50; however, halogen light produced the highest CRI ratings.

**Fig. 6:**
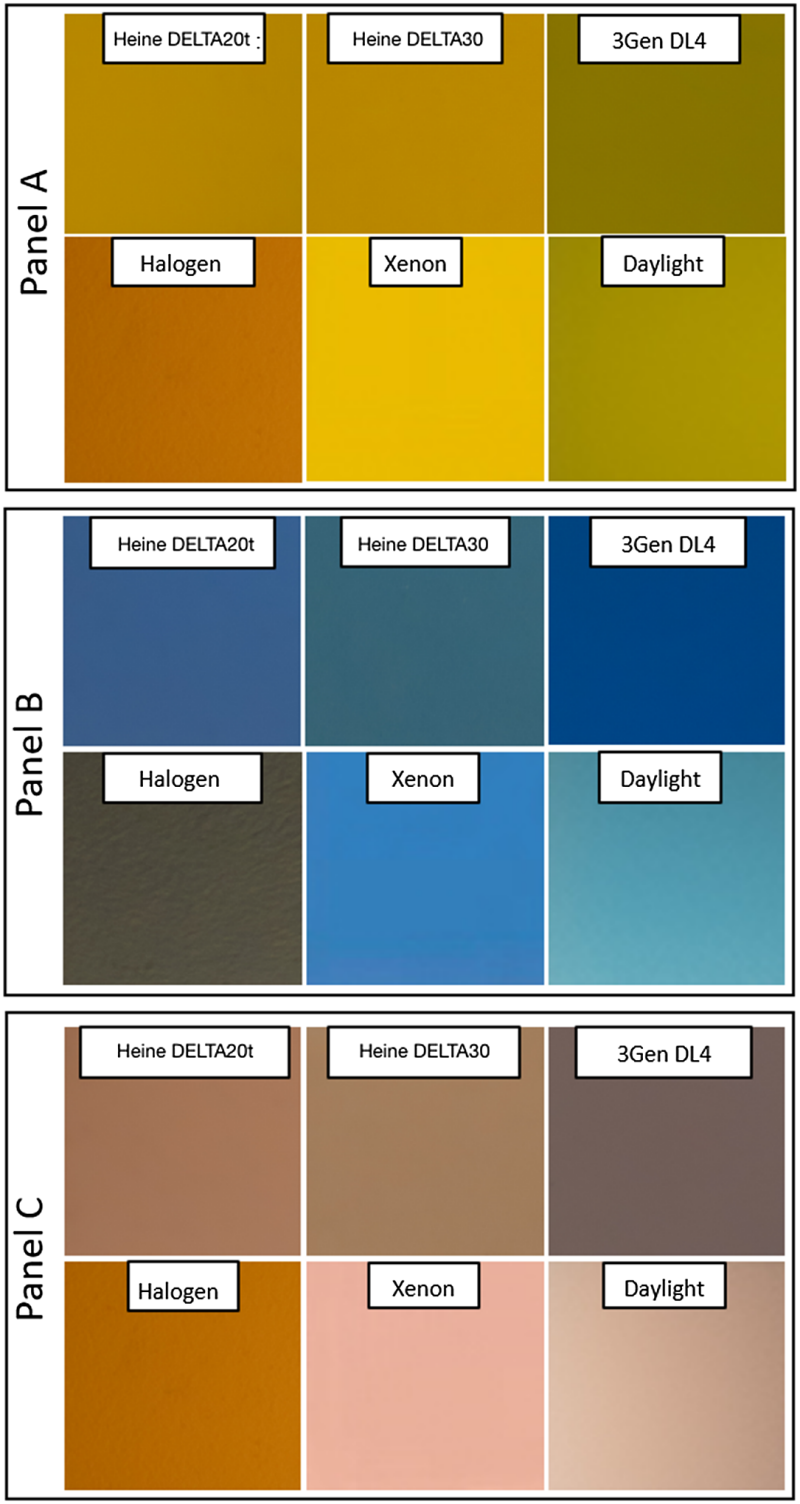
ColorChecker™ images set to 5000 K of three select colors with variations of illuminant CCT. Standard acquisition settings and uncorrected white balance were used to photograph three colors from a micro ColorChecker under six distinct illuminants. Using an iPhone 11, the same exposure was made with different devices showing how panel A (light skin), panel B (blue sky), and panel C (orange–yellow) appear differently under each source. By setting the color balance to 5000 K, we demonstrate how far off the colors might look, uncorrected, to a camera. Matching the CCT to the illuminant (color correction) would allow for a more accurate rendition, akin to human visual systems.

ColorChecker images of three color patches demonstrated noticeably different results when acquired under each light source (Fig. 6). The images were placed side-by-side, so differences could be appreciated visually. ColorChecker images were not color corrected, giving xenon the ability to most accurately render the color at 5000 K.

ΔE was calculated following exposure and color correction in L*a*b* color space for two LED dermoscopes. The range is from 1 to 100 with 1 corresponding to nearly imperceptible and 100 indicating opposite colors. Values measured showed perceptible differences of varying degrees. For the color “light skin,” dermoscope #3 compared with dermoscope #4 showed ΔE=3, which is a barely noticeable difference. For the color “blue sky,” ΔE=6, demonstrating a more pronounced difference. For the color “orange-yellow,” ΔE=8.

**Fig. 7:**
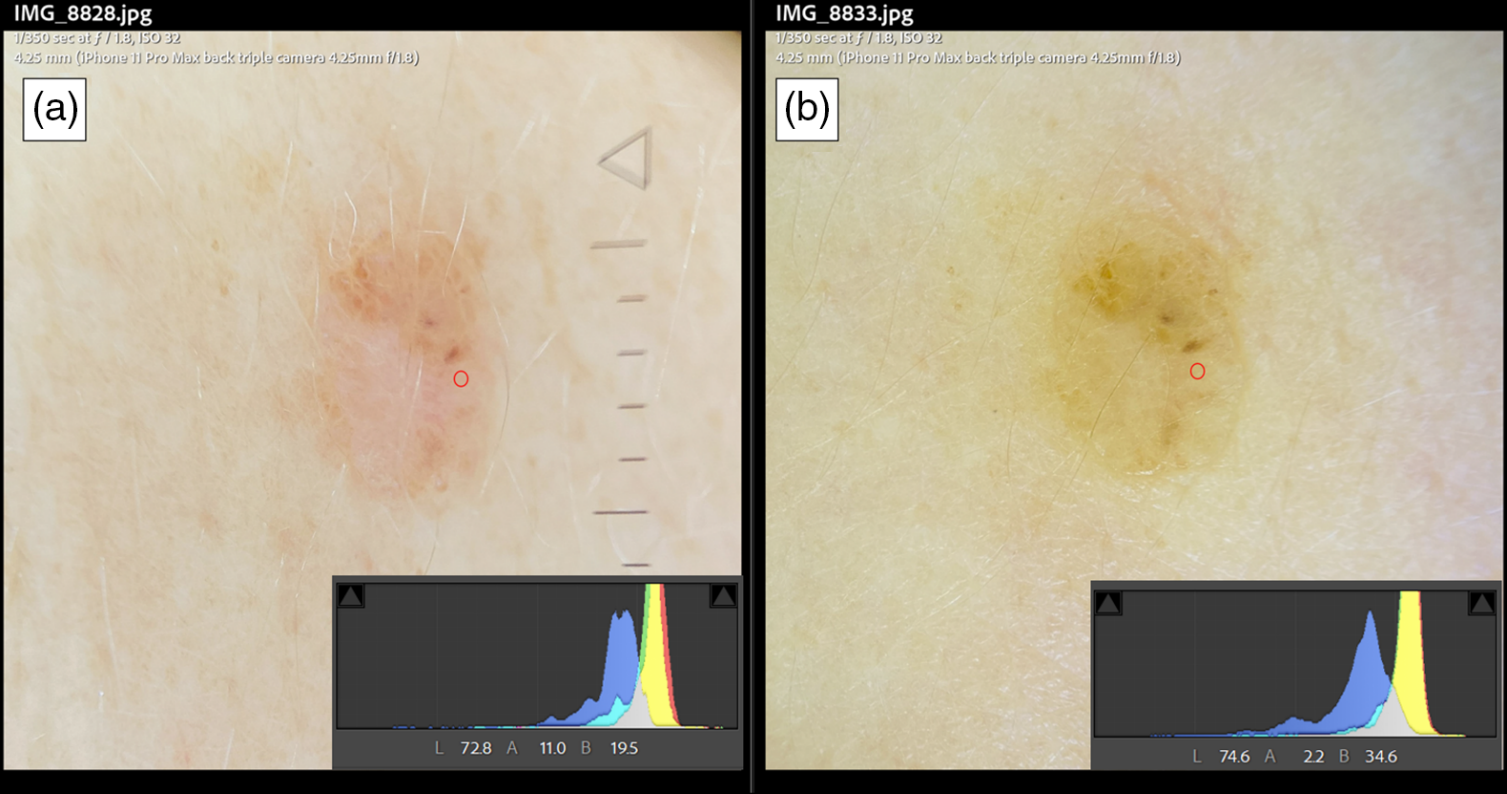
A real-world scenario of differences in dermoscopes LED color tint showing the same pigmented lesion and standard exposure settings. Panel A is acquired using a dermoscope with a slightly pink shift using an iPhone 11 with its corresponding histogram and a point localized on the lesion with a red circle and the corresponding L*a*b* values. Panel B using a different dermoscope with more of a yellow hue, its corresponding histogram, and a red circle in the same localization showing L*a*b* values of this point.

These ΔE are small because the images were corrected prior to calculating. They represent visually perceptible differences and, without color balance, can persist to an even greater degree in images acquired using different dermoscopes in practice. To demonstrate what this difference might mean practically, in a clinical scenario, an image of a dermal nevus was captured with two dermoscopes with the same acquisition and exposure settings (Fig. 7). The two images show visible color differences; when L*a*b* values of the same region in both images were compared, ΔE=14.5.

The results illustrate several concepts with regard to spectral coverage, the most apparent being that LEDs in different manufactures’ dermoscopes do not match one another. We measured two other devices from 3Gen (not shown), and they appeared to have consistent SPDs. Dermoscope #3 had a higher CRI of R9 (and other colors) as well as higher TM-30 fidelity ratings, giving it better color matching and rendering ability than all of the dermoscopes tested when referenced to D50.

Emission light interacts with chromophores contained in skin and is returned to either our eye or a camera’s sensor. Our results demonstrated, that when using a common mobile camera with identical settings, the same color appeared differently under each dermoscope as well as across reference sources. Our results indicated that the degree of difference between reflected colors acquired with two popular LED devices was indeed perceptible. The differences were within the range of three to eight, which is considered visually noticeable. ΔE was measured after white balancing, similar to what occurs in practice with in-camera auto white balancing.

## 4 Discussion

Consistent illumination in the field of dermatology and dermoscopy is important for evaluation during visual diagnostic exams and interpretation of the resulting images. Although there is not one specific “correct” light for skin visualization, color consistency may be underrecognized for its importance to interpretation. Considering the importance of color to dermatology and skin investigations, it is somewhat surprising that there is limited research into optimal exam lights. Surgical lighting has more studies related to optimization for viewing and imaging of specific tissues and applications;^24^^,^^47^^–^^50^ albeit no universally agreed upon type of light has been identified. Similar to dermatology, surgical lighting that used to rely on halogen bulbs is being replaced with “white” LEDs. Despite similar lighting consistency challenges between fields, surgery demands more control and accuracy than normal overhead incandescent (tungsten) and fluorescent bulbs are capable of providing. Although most dermoscopy devices use “white” LEDs, dermatology exam rooms are often lit with overhead fluorescent or incandescent bulbs. Unless a window with natural sunlight is present to increase spectral coverage, or a sufficient continuous broad spectrum artificial light is employed, large gaps in spectral distribution may hinder the practitioner’s ability to identify subtle findings with color features, an issue that may be of particular relevance to skin of color examinations.

Human visual systems can account for some of the variability in lighting and color more efficiently than imaging systems; however, a thorough discussion is beyond the scope of this review. The maximum efficacy or peak sensitivity is around 555 nm, in the green region of the visible spectrum. It is thought that the evolution of humans’ visual perception of color is not driven by maximum light intensity, but rather by the optimal wavelength at which information can be obtained about the environment.^51^ With this in mind, future LEDs may close the “green gap,”^52^ thereby increasing the available information for skin examination. Artificial light, LEDs included, typically intends to render its environment as accurately as possible. Metamerism occurs when two distinct chromophores appear as the same color under one light source but not under another, as is the case of pigment and blood overlapping visually when viewed under one dermoscope but not when viewed under a different one. One explanation for this phenomenon is that the biochemical properties of melanin and hemoglobin absorb/reflect light differently across the visible spectrum. On one end of the spectrum, melanin reflects more light than blood, whereas, at the other end of the spectrum, blood is more reflective. Melanin reflects light evenly, with a linear increase, but hemoglobin has a sharp spike in its reflectance. The “green gap” characteristic of many white LEDs means that the energy around 500 nm is quite low, such that color separation here could be challenging. Furthermore, the areas with sufficient spectral coverage from LEDs, approximately between 540 and 620 nm, are indeed where blood and melanin overlap and crosstalk between the red and brown chromophores is higher. This means that the chromophores reflecting red and orange as distinct colors are illuminated with low energy in the parts of the spectrum where their separation is most pronounced and are illuminated with substantially more power in the spectral region of overlap, to a greater or lesser degree depending upon the exact SPD of the LED used (Fig. S3 in Supplemental Material).

We have demonstrated that the color variations between dermoscopes is wider than is considered acceptable for other similar applications and that different lights used in dermatology produce distinct reflected colors. Although we recognize the value of introducing specialized LEDs (such as orange or UV) for visualizing specific skin findings, the lack of consistency in the “white” LED characteristics is troublesome. It is unlikely that these color variations lead to blatant misdiagnosis; however, color fidelity and reproducibility is an important feature for AI-based diagnostic algorithms. Kelvin temperature (CCT) has been recommended as a DICOM data element, presumably for this very purpose.^16^ This inclusion of CCT to image metadata may allow for increased ability to correct color when database construction occurs; however, as we have demonstrated, a significant degree of difference persists even in the context of white balance correction.

We know that melanin possesses distinct optical properties at different depths, such as the Tyndall effect seen with deeper pigment appearing more blue. These subtle differences are important for understanding skin biology, biophysics, and pathology. The optical properties of human skin vary widely in the context of different skin types and diseases, making color variation of particular interest in cutaneous biophysics. The absorption and scattering events that take place as light is transported and reflected are complex but can be modeled with some degree of confidence using computational methods.^53^ Accounting for variations of LED color may hinder the ability to model light transport in skin.

It appears that the CRI of reds, specifically R9, is quite low for LEDs; differences noted in the reds are of particular concern to cutaneous imaging. Melanin and blood can sometimes be challenging to differentiate under dermoscopy, and this issue is likely further complicated by the lack of color rendering consistency. Slight differences between violet, pink, rose, red, and brown are altered when using different dermoscopes and light sources. Fig. 7 demonstrates this issue with a real-world clinical image. This comparison illustrates the subtle differences between dermoscopes LED tint; however, it is somewhat limited by the lack of a “ground-truth” measurement, an experiment that may be appropriately performed with a spectrophotometer, which is a potential area of future research.

LED technology has advanced tremendously in recent years and continues to evolve at a rapid pace. Dermoscopy device developers may consider standardizing LED type(s) or, at the bare minimum, having technical specifications, such as SPD/CCT, made available to dermatologists and clinicians who use the products. Some awareness of potential pitfalls in the interpretation of images resulting from different dermoscopes is appropriate because the reflected color is altered according to the wavelengths of light’s absorption and scattering events with chromophores contained in skin. AI and ML algorithms using dermoscopic image data that hinge on color as a feature may benefit if the lighting is standardized in the field of dermoscopy.
