# Supplementary material for: Dermoscopy and skin imaging light sources: a comparison and review of spectral power distribution and color consistency
Source: J Biomed Opt. 2022 Aug 8;27(8):080902. doi: 10.1117/1.JBO.27.8.080902 (PMC9360608; doi:10.1117/1.JBO.27.8.080902)

Supplemental Figure 1: Composite of original, RAW image files of micro ColorCheckers through different dermoscopes showing exposure settings. The acquisition settings were standardized and DNGs were acquired. The DNG (RAW) files may have their color temperature and exposure altered in the computer during post processing (unlike jpeg). The DNG images were cropped and color temperature was set to 5000K for the side-by-side comparisons in Figure 6.

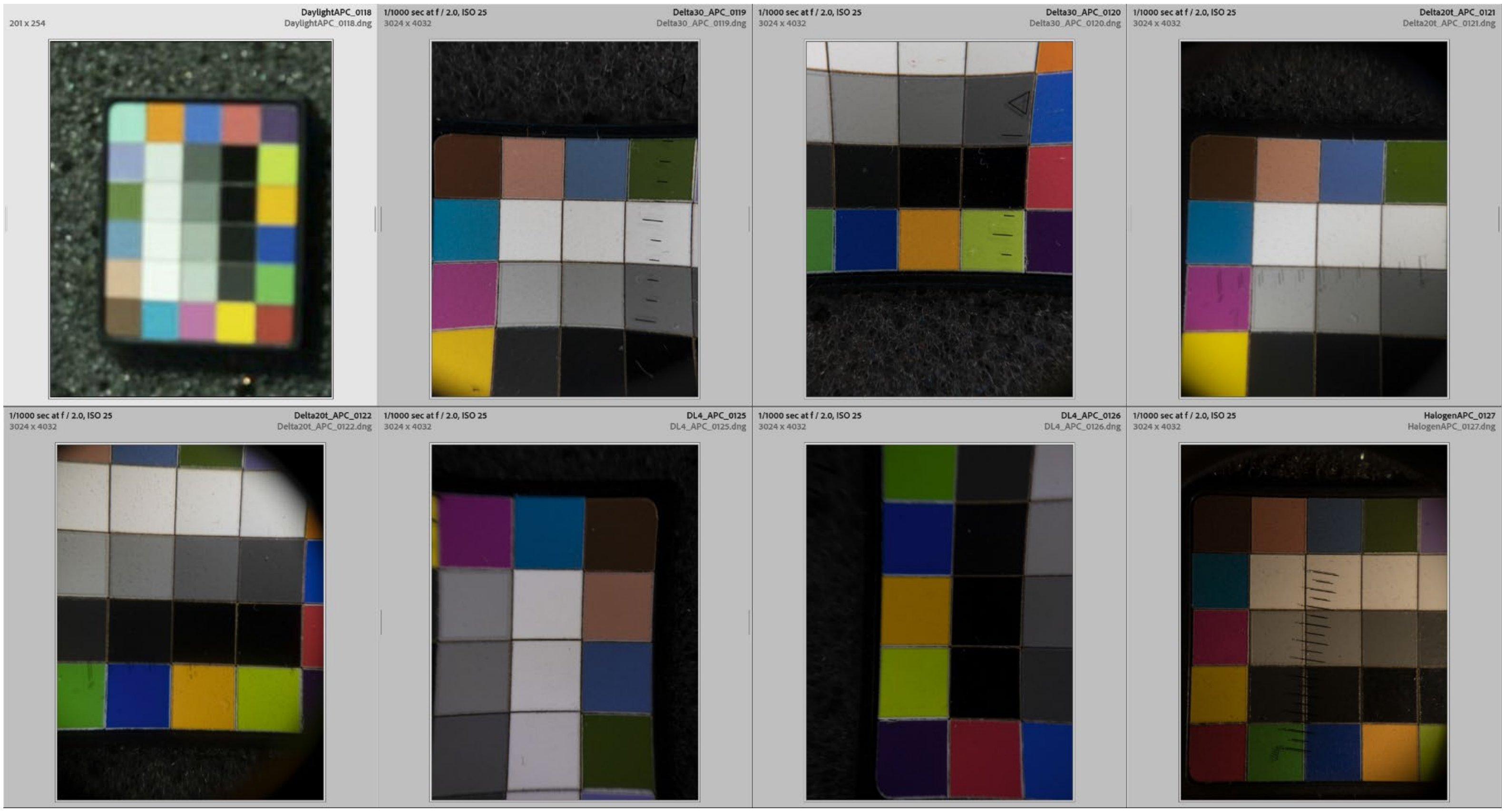

Supplemental Figure 2

ΔE Formula: CIEDE2000

$$\Delta E_{00}^{*} = \sqrt{\left(\frac{\Delta L'}{k_L S_L}\right)^2 + \left(\frac{\Delta C'}{k_C S_C}\right)^2 + \left(\frac{\Delta H'}{k_H S_H}\right)^2 + R_T \frac{\Delta C'}{k_C S_C} \frac{\Delta H'}{k_H S_H}}$$

$$\Delta L' = L_2^* - L_1^*$$
$$\bar{L} = \frac{L_1^* + L_2^*}{2} \quad \bar{C} = \frac{C_1^* + C_2^*}{2}$$
$$a_1' = a_1^* + \frac{a_1^*}{2}\left(1 - \sqrt{\frac{\bar{C}^7}{\bar{C}^7 + 25^7}}\right) \quad a_2' = a_2^* + \frac{a_2^*}{2}\left(1 - \sqrt{\frac{\bar{C}^7}{\bar{C}^7 + 25^7}}\right)$$
$$\bar{C}' = \frac{C_1' + C_2'}{2} \text{ and } \Delta C' = C_2' - C_1' \quad \text{where } C_1' = \sqrt{a_1'^2 + b_1^{*2}} \quad C_2' = \sqrt{a_2'^2 + b_2^{*2}}$$
$$h_1' = \text{atan2}(b_1^*, a_1') \mod 360°, \quad h_2' = \text{atan2}(b_2^*, a_2') \mod 360°$$

$$\Delta h' = \begin{cases} h_2' - h_1' & |h_1' - h_2'| \leq 180° \\ h_2' - h_1' + 360° & |h_1' - h_2'| > 180°, h_2' \leq h_1' \\ h_2' - h_1' - 360° & |h_1' - h_2'| > 180°, h_2' > h_1' \end{cases}$$

$$\Delta H' = 2\sqrt{C_1' C_2'} \sin(\Delta h'/2), \quad \bar{H}' = \begin{cases} (h_1' + h_2' + 360°)/2 & |h_1' - h_2'| > 180° \\ (h_1' + h_2')/2 & |h_1' - h_2'| \leq 180° \end{cases}$$

$$T = 1 - 0.17\cos(\bar{H}' - 30°) + 0.24\cos(2\bar{H}') + 0.32\cos(3\bar{H}' + 6°) - 0.20\cos(4\bar{H}' - 63°)$$
$$S_L = 1 + \frac{0.015\left(\bar{L} - 50\right)^2}{\sqrt{20 + \left(\bar{L} - 50\right)^2}} \quad S_C = 1 + 0.045\bar{C}' \quad S_H = 1 + 0.015\bar{C}'T$$
$$R_T = -2\sqrt{\frac{\bar{C}'^7}{\bar{C}'^7 + 25^7}} \sin\left[60° \cdot \exp\left(-\left[\frac{\bar{H}' - 275°}{25°}\right]^2\right)\right]$$

Supplemental Figure 3: Overlay of melanin/hemoglobin reflectance with LED spectral power distribution. Separation of brown and red may be challenging in the regions that have low energy illumination from the LED such as in the blue-greens and deep reds.

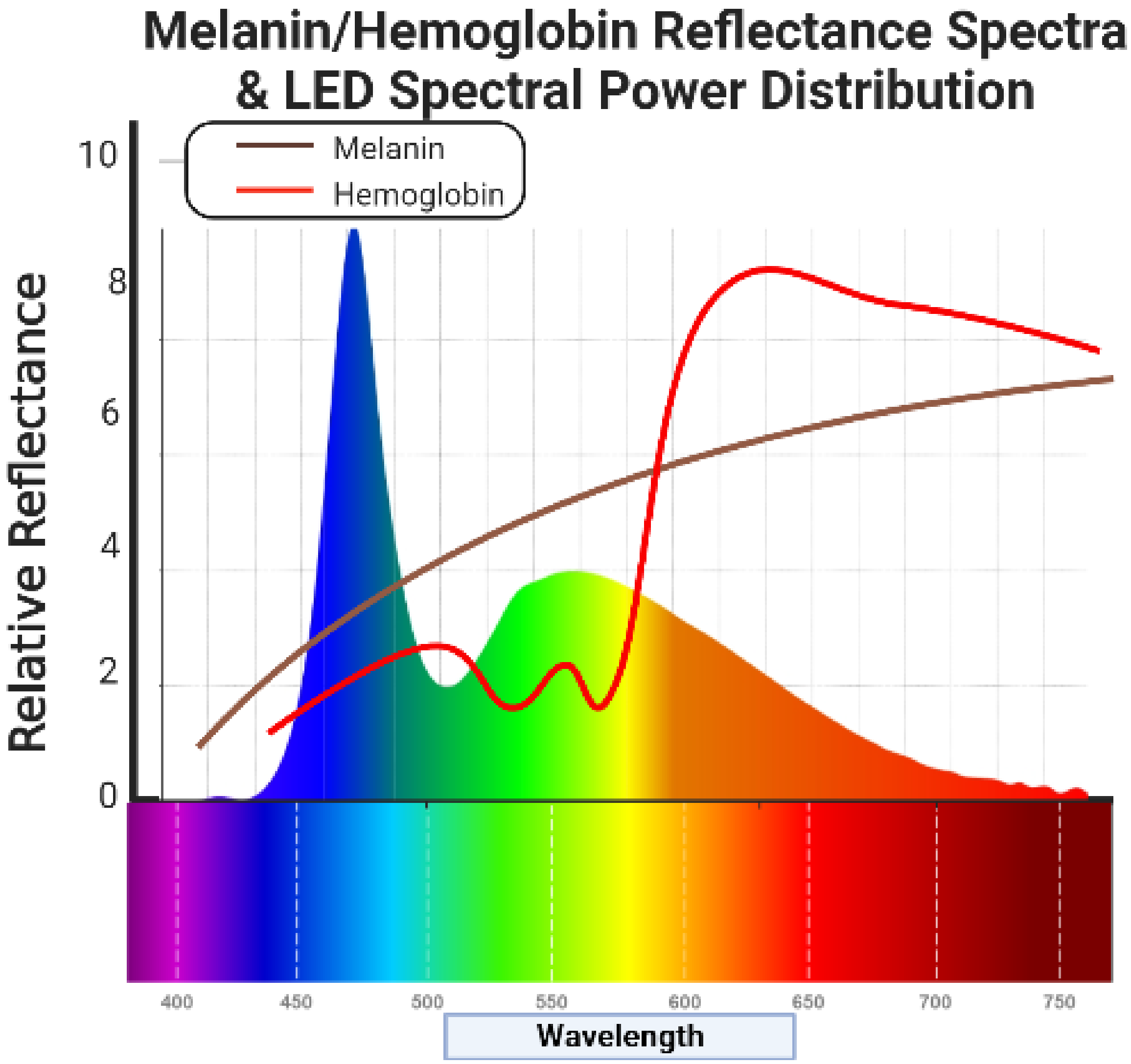

Supplement: Supplementary file 1 [file JBO_027_080902_SD001.pdf]
